# The Determinants of Private Tutoring Participation for Mathematics in China: Focusing on the Role of Student Metacognition

**DOI:** 10.3389/fpsyg.2020.00603

**Published:** 2020-04-06

**Authors:** Yuhuan Zhang, Xiao Ma, Lidong Wang

**Affiliations:** ^1^School of Mathematics and Statistics, Henan University, Kaifeng, China; ^2^Collaborative Innovation Center of Assessment for Basic Education Quality, Beijing Normal University, Beijing, China

**Keywords:** family SES, metacognition of mathematics learning, moderating effect, private tutoring, China

## Abstract

Participation in private tutoring has been a global educational phenomenon and is especially prevalent in China. The present study conducted a student-report questionnaire and collected school reports of mathematical achievement to track a year of the longitudinal variation of private tutoring and obtained an analysis of the impact of metacognition on private tutoring participation and its moderating effects between environment-related and initial indicators and mathematical private tutoring participation. The empirical results of this study showed that metacognitive level for learning process monitoring, family economic condition, and mathematics achievement could impact the decision to participate in private tutoring. The results also showed that family economic condition could moderate the effect of metacognitive level for learning process monitoring on the decision to participate in private tutoring. Practical implications for educational competent department and the schoolteachers were discussed.

## Introduction

Taking PT after class to complement in-school learning, also known as shadow education, is a global education phenomenon (Davies, 2004; Mori and Baker, 2010; Guill and Bos, 2014; Byun et al., 2018). It has long been practiced as individual tutoring on an informal basis and has become a multi-billion-dollar global service-industry (Byun and Baker, 2015; Byun et al., 2018). Students in South-Eastern and Eastern Asian countries are more likely to pursue shadow education than their counterparts in many other regions (Byun et al., 2018). Data from the Chinese National Assessment for Education Quality, an authorized national education-oriented survey, documented that 43.8% of fourth grade and 23.4% of eighth grade students participated in mathematics shadow education in 2015, and approximately 45% of them engaged in PT outside school for more than 2 h per week (National Center of Education Quality, 2018). Scholars from different communities have talked about this issue from diverse points of view, including the effectiveness of PT on student achievement (e.g., Zhang, 2014), political discussion of PT (e.g., Bray and Lykins, 2012), the social study of PT (e.g., Cheng and Chen, 2014) and the determinants of PT (e.g., Liu, 2012). Of these, the determinants of PT participation have been among the fundamental issues (Byun et al., 2018), if the families actually use the PT properly, which could help to “earn their payment back.”

### Determinants of Participation in Shadow Education

Several previous studies have discussed the factors that impact demand for PT based on different cultural settings, different conceptual frameworks, and different statistical models. As stated in the introduction, it is well established that students in Asia spend much time pursuing academic achievements (Bray and Lykins, 2012; Byun et al., 2018). They engage in crowded curriculum schedules oriented toward numbers and literature starting at an early age and report a high proportion of PT participation (Bray and Lykins, 2012).

This unique social-educational phenomenon is considered as the consequence of education being traditionally rewarded and the fierce competition caused by the dense population (Wang et al., 2014). Existing studies have described the features and characteristics of PT and the students who participate in it. Both contextual and social-psychological variables were used in prior studies (Choi and Park, 2016). These studies have mainly focused on family related factors with respect to family decision making, including the indicators of total family income, father’s income, and parents’ education level (e.g., Xue and Ding, 2009; Kim and Park, 2010; Liu, 2012). Data from a Chinese nationwide survey suggested that family SES was positively associated with PT participation and the relevant expenditures (Xue and Ding, 2009). Another study showed that the education level of the mother was a remarkable indicator (Cheng et al., 2012). Similar results were also reported in South Korea with positive results for social setting, household income, and parents’ educational level (Kim and Park, 2010). However, inconsistent results were also reported, especially from different cultural settings. Another study reporting logistic results from Taiwan of China suggested that students tended to take PT across family SES as represented by the family income or father’s occupation, which implied that the need of attending cram schools was easy to meet in Taiwan of China (Liu, 2012).

Other than fundamental characteristics of families (parents), further comprehensive variables were discussed. Evidence from Canada found a connection between parents’ involvement with the children and relevant satisfaction levels, such as the parents’ satisfaction with public school (Davies, 2004). Liu and Bray (2018) applied a mixed method and found that factors influencing parental choices included not only cost and the availability of time but also the children’s academic performance, their stage in schooling, and educational system reforms, with a focus on consumer preferences. That indicated how parents rank different possibilities based on the perceived utilities of those possibilities in supporting their children’s education.

In addition, some studies proposed that students from schools with a higher student–teacher ratio than their peers from schools with a lower ratio was positively associated with PT participation and the relevant expenditures (Zhang, 2014). Another survey suggested that school background, a variable given little attention, was positively associated with PT participation and the relevant expenditures (Xue and Ding, 2009).

The personal characteristics of students that could also potentially impacted the dynamics of decision making for participating in PT were rarely discussed. One exception was student achievement; potentially, tutoring represents a relatively affordable strategy for a variety of parents who aim to improve their children’s performance (Davies, 2004). Several studies have explored and found diverse relationships between mathematics achievement and PT. Baker et al. (2001) found that the modal strategy for using shadow education was for remedial purposes (i.e., negative, statistically significant coefficients for the relationship between student achievement score and shadow education use) in over three fourths of 41 TIMSS countries studied, including Denmark, Germany, and the U.S. However, Zeng and Zhou (2012) used a large-scale dataset from Beijing and found a significant positive correlation between participation in shadow education and improved performance among eighth grade students and no significant correlation among fourth grade students. Parents might select PT not because their children actually need supplementary learning to enhance their low achievement but to maintain their children’s advantage when other parents select PT. This indicated that the circumstances in a Chinese setting might be different from those in western cultural settings (Zhang, 2014).

The majority of the samples in previous studies focused on students at the primary and second school levels who seemed to be passive learners constrained to this extra learning under the decisions of their parents. In fact, students, especially those in senior grades, adopted appropriate learning strategies for academic improvement according to their own learning processes and results. For them, there is a lack of understanding of the contribution of students themselves in choosing PT as an alternative academic improvement strategy and in the underlying decision-making process, especially for older students (e.g., high school level).

For the empirical methodology, logistic analysis was typically used to sufficiently show the relationship between several factors and PT. Many quantitative studies viewed the demand for private tutoring in terms of snapshots of particular moments, and these snapshots were generally of different cohorts of families and students rather than longitudinal surveys of the same families and students (Liu and Bray, 2018); this may mean that previous results have been biased. Therefore, longitudinal studies are a necessary research direction going forward. Moreover, most previous studies employed a subscale from a questionnaire or several questions from national education surveys instead of generating a specific measurement to describe PT participation behaviors and indicators, including facilitators and barriers.

### The Theoretical Analysis of Determinants of PT Participation

Although there is a growing body of research that examines the determinants of PT, few studies have extensively and explicitly discussed theories behind the decision-making processes of parents and students regarding the use of PT (Byun et al., 2018). However, statistical analysis of determinants of PT participation is fundamentally important.

Previous studies generally concentrated on environmental factors, including social background and school characteristics [such as the student–teacher ratio and the loading of curriculum (Zhang, 2014), teacher characteristics such as teacher power (Zhang, 2014), and family characteristics such as the SES of families (Liu, 2012)], which influence participation in PT. The backgrounds of the researchers, who come from educational policy, educational sociology, and educational economics, could partly explain this.

A reproduction model of family decision making highlights that elite families seek shadow education for advanced educational opportunities to reproduce their elite status for their children (Stevenson and Baker, 1992) from the perspective of educational sociology, which predicted that shadow education is an important strategy for high SES parents to enhance their children’s academic success (Byun et al., 2018).

The concept of school choice provided a conceptual framework for the analysis of PT selection. This concept is more obviously applicable to the college choice industry (McDonough, 1994), which refers to parents seeking strategies to enhance their children’s competitiveness, whether at the advanced or remedial level, to gain entrance to specific colleges (Davies, 2004). Davies (2004) regards PT as a default consumer choice in schooling, which is similar to the choice for private schooling and homeschooling (in the Canadian background).

However, few studies discussed if the families’ use of PT were proper, such as if the decision actually benefit the students, and if the decision based on the diagnosis on students’ learning or the needs of mathematical learning.

### Metacognition of Mathematics Learning and PT Participation

To faster a better academic performance, PT seems a particular strategy for students to reinforce their knowledge, enrich their diagram, and prepare particular advance organizer for further studies. Therefore, learners decide to take part in according to their experience of previous learning and their judgment from task difficulty, online monitoring of their performance, feelings of knowing, and confidence judgment. However, the existing studies paid little attention on discussing the underlying mechanism from the perspective of psychology of learning and even less so than the role of metacognition in the conceptual framework of factors that influence participation in PT.

The understanding of metacognition varied among researchers (Alexander et al., 2003), but typically, it is defined as a higher-level cognition that provide knowledge about cognition and control of cognition (Flavell, 1979). Evidence from educational contexts show that learners metacognition include their person knowledge, task knowledge and strategy knowledge (Flavell, 1979), which help learners to aware and monitor their learning process and results to an ongoing attempt to plan, check, monitor, select, revise, evaluate, etc, which lead to an improvement of academic performance on mathematical skill (Garofalo and Lester, 1985). There is a considerable impact from metacognition on mathematics performance, through sharing about 15-20% of common variance (Schneider and Artelt, 2010). Pintrich et al. (2000) suggested a 4-components model of metacognitive awareness and monitoring, including ease-of-learning judgment, judgments-of-learning, feeling of knowing judgment and confidence rating. It highlighted the important role of metacognition in providing knowledge about when and how to use particular strategies for learning or problem-solving (Flavell, 1979; Schoenfeld, 1992; Panaoura and Philippou, 2003). Some existing studies show that metacognition monitors and evaluate the whole process of mathematical learning (Wang et al., 2016), including, for example, planning a clear and definite learning objective (Cui et al., 2018), and assessing one’s own capabilities and limitations in this domain in general (Garofalo and Lester, 1985). It could potentially play an important role in the decision-making process of PT selection.

There is little studies investigate the impact of metacognition on PT participation. A line of studies on school choice show that consumer choice in education is a complex process (Davies, 2004), and existing studies might not comprehensively reflect the construct of determinants of PT (Byun et al., 2018). In addition, previous studies were generally limited to a compulsory level of education and tended to focus only on family decision-making (e.g., Davies, 2004; Liu and Bray, 2018), in particular the decision-making of parents for young children. As they approach adulthood, however, high school students tend to have much more autonomy than compulsory pupils do.

Regarding the role of metacognition in learning and the limitation of previous conceptual framework of factors that influence participation in PT, we hypotheses that students’ metacognition, especially focusing on the whole process of learning, play an important role in PT participant. The current study propose a more comprehensive model that integrated students’ metacognition on mathematic learning process into the conceptual framework of factors that influence participation in PT. It is expected to analyze the characteristic of students’ learning, which tend to relate to the metacognition of (mathematical) learning.

### The Current Study

Previous studies tend to describe decision-making regarding PT participation from the parents’ point of view (Liu and Bray, 2018), but understanding the structure of the determinants of PT participation from high school students themselves may also contribute toward revealing the underlying decision process for taking PT as a supplement to in-school learning. The current studies mainly focus on the contribution of student metacognition in mathematical PT learning taken among a cluster of social- and family related indicators.

It could be hypothesized that high school students who had gained more metacognitive information would be more involved in making decisions regarding whether to choose PT, based on their understanding of their own learning limitations. Consequently, students’ understanding of learning (metacognition of mathematical learning) would potentially related to their decision to participate in PT. For example, students’ metacognitive abilities would help them to readily detect the need to select PT in the case that they could clearly tell that high school mathematics is difficult or challenging for them.

Moreover, it could be hypothesized that if the family economic condition was high but mathematics achievement was low, the probability of choosing PT would be higher, which would indicate a moderating role of the two variables.

This could then explain the structure of the determinants of PT participation from the view adopted from psychology of learning, which focuses on individual internal characteristics beyond just social context or convenience variables, often the limited focus of large-scale surveys.

**FIGURE 1:**
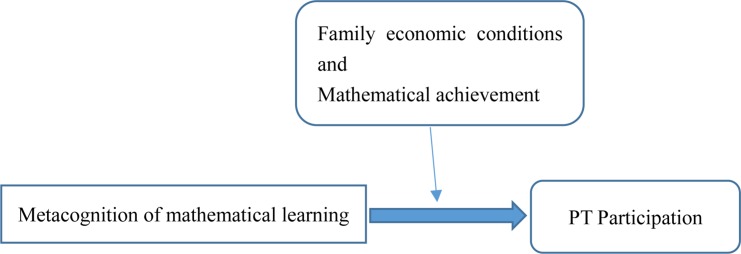
The theoretical framework.

The present study aims to reveal the indicators of PT participation in high school students and develop a specific theoretical framework integrating the environmental and initial indicators of PT participation. From the perspective of educational sociology, family economic condition and student mathematics achievement were the two fundamental factors that could predict PT participation; metacognition was employed as the essential measurement to describe the initial student indicators and could be moderated by the two fundamental factors above. Details were illustrated in Figure 1.

The theoretical framework proposed by the present study is a multi-domain framework that integrated family related indicators and individual indicators into a complete system for understanding the mechanisms of PT participation. Choosing PT is a dynamic procedure that might vary at different points of the high school timeline. Decisions may also need to be made multiple times, for example, at the beginning of every school year or school semester. Therefore, a longitudinal design was conducted to illustrate the differential structures of PT determinants.

As the literature discussed above, it is expected that this multi-domain theoretical method would help to explore the interaction between environmental and initial variables and PT participation, especially the role of metacognition of mathematical learning. If the metacognition of mathematical learning could predict the PT participation, it might imply that the decision-making of PT participation was proper, which based on the actual need of extra tutoring of mathematics, not merely under the social pressure. The two hypotheses to be tested in the current study are as follows:

H1: Declarative metacognition for mathematical learning has an impact on PT participation when paired with environmental indicators and individual indicators, such as family economic condition and mathematic achievement.

H2: Metacognitive level has a moderated effect on PT driven by both environmental and individual indicators. This suggests that students with a certain level of metacognition would more likely decide to participate in PT relative to students classified by factors such as their family economic conditions and mathematical achievements.

## Materials and Methods

### Participants and Procedure

Data was obtained in November 2018 using self-reported questionnaires. Students in the 11th grade from one large high school in Kaifeng, a typical medium-sized city in China, were sampled using the cluster sampling method. Eight hundred ninety-two of 915 mathematics questionnaires were returned, then subjected to the data cleaning progress. Any missing instance variables were marked and excluded from the final data analysis. Therefore, there were a total of 667 participants (43.8% male, 56.2% female; 438 took the science track, and 229 took the humanities track at the end of the first semester in the 10th grade). Students completed the questionnaires in class with the requirement and encouragement of their school teachers. A cover letter was set in the first page of the questionnaire to invite students to participate and explain the background of the survey. Four trained graduate students of mathematics education worked on distributing, monitoring, and collecting the data.

The students in ordinary class have about 1 day per week to rest, and the students in the key classes would have less rest time each week. The students had nearly 2 months of vacation time during the summer vacation. This information suggests that the sample students tended to have a heavy learning load, as the PT is done during their limited time outside school.

### Measures

#### Mathematics Achievement and PT Participation

**FIGURE 2:**
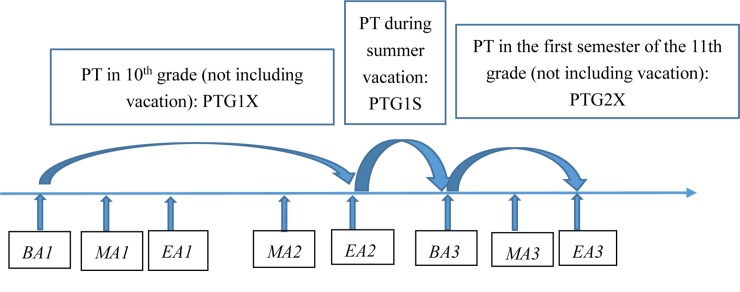
The data collection process. *BA*: a mathematics achievement score nearly at the beginning of the semester. *MA*: a mathematics achievement score in the middle of the semester. *EA*: a mathematics achievement score at the end of the semester.

The mathematics achievement variables came from the school reported mathematics test scores, aligning with the actual learning progress of mathematics of the sample school. The full credit were 150. This data provided a series of mathematics test scores for each student starting from the beginning of the 10th grade and extending until the end of the first semester of the 11th grade (including winter and summer vacations, as showed in Figure 2). This follow-up data provided the possibility to explore the dynamics of the PT participation decision-making procedure for students adapting to a new learning environment.

The dependent variable is the participation of PT in mathematics during summer vacation after the 10th grade (1 = yes, 0 = no): PTG1X: PT in the 10th grade [not including vacation], PTG1S: PT during summer vacation.

#### Students and Family Characteristics

For this study, family economic status income were used to characterize family SES. The high school students response the rubrics that Family economic status (1 = very poor, 2 = relatively poor, 3 = average, 4 = relatively rich, 5 = very rich), which could reflect their understanding of their family SES and impact their decision-making of PT participation.

The gender of students was used as the control variable: Gender (1 = male, 2 = female).

#### The Metacognition of Mathematics Learning

Declarative metacognition of mathematics, including ten rubrics selected from a validated questionnaire (Wang et al., 2016) as in Table 1, focusing on the three sub-domains: sm1–sm10. These rubrics focused on monitors and evaluating the whole process of mathematical learning (Wang et al., 2016), including, for example, planning a clear and definite learning objective (Cui et al., 2018), and assessing one’s own capabilities and limitations in this domain in general (Garofalo and Lester, 1985). Each of them reflected one kind of aspect of students’ status in metacognition ability level, and did not have a high level of correlation. All the ten rubrics were applied in the finial model, and did not need to combine them into one variables. It could detailed implied the mechanism of decision-making process.

**TABLE 1:** Rubrics related to the whole mathematics learning process.

The following 10 5-point Likert rubrics identified the metacognition of the whole learning process
sm1: I can clearly assess my mathematical learning and identify my class rank in mathematics learning capacity.
sm2: I can clearly tell if high school mathematics is difficult or challenging for me.
sm3: When learning different types of mathematical knowledge, I will set my learning objective accordingly. For instance, I will enhance my spatial imagination capacity in solid geometry study.
sm4: My estimate of my scores on mathematics tests is very close to the actual scores.
sm5: I feel a sense of accomplishment, satisfaction, and excitement when a mathematics problem is successfully solved.
sm6: I can clearly state which parts of high school mathematics I have blind spots in. For instance, I cannot accurately explain the concept of skew lines, or I cannot distinguish negative propositions from the negation of propositions.
sm7: I can clearly assess my capacity for learning different types of mathematics knowledge. For instance, I know whether I have a firm grasp of trigonometric functions or solid geometry.
sm8: I often lack self-confidence before learning new mathematics knowledge.
sm9: After learning mathematics for a while, I will do self-assessments of my learning outcome.
sm10: I often do self-examination to figure out which aspects of my mathematics knowledge I have not mastered well.

*The rubrics sm1, sm6, and sm7 are about mathematics metacognition knowledge; sm2, sm5, and sm8 are about mathematics metacognition experience; and sm3, sm4, sm9, and sm10 are about mathematics metacognition monitoring.*

### Analytical Strategy

In the review of previous studies, to analyze which factors affect the probability of PT participation, the binomial logistic regression was used. Let *P* refer to the probability that students will participate in PT; 1–P refers to that for those who do not, X*_ki_* refers to the independent variables, such as the metacognition of mathematical learning and students’ mathematical achievement, β_*k*_ refers to effect size, and α refers to the constant of the model. The function form, adapted from Xue and Ding (2009), is shown as follows:

$$\text{ln}\,\left(\frac{P}{1-P}\right)=\alpha +\sum_{k=1}^{K}\beta_{k}\,X_{k\,i}$$

Many quantitative studies viewed the demand for PT in terms of snapshots of particular moments, and not in terms of longitudinal examination of the same families and students (Liu and Bray, 2018). We also found that existing studies were based on cross sectional data, and PT participation was not previously included in the model. In our studies, we traced the students’ PT participation for about a year and a half (though only through one survey) and could deeply understand the dynamics of PT participation. We conducted the following two analyses based on data from one sample and one sub-sample (science-track) in different time-points.

Analysis I: The determinants of all students’ participation in PT during the 10th grade school year.

Analysis II: The determinant of science-track students’ participation in PT during the summer vacation of the 10th grade.

## Results

### Descriptive Statistics

Tables 2, 3, show the descriptive statistics for the high school sample in analysis I and analysis II. For analysis I, about 44% of our participants were male, and 36% of these males participated in PT in the 10th grade (not including vacation). Their average score at the beginning of the first semester of the 10th grade was 100.69 (total score: 150). For analysis II, about 55% of our participants were male, and among them, 31% participated in PT in the 10th grade (excluding vacation) and 34% participated in PT during summer vacation, which indicated that a relatively high participation rate was maintained compared with that of the primary and secondary level. The average score at the beginning of the first semester of the 10th grade was 94.58.

**TABLE 2:** Descriptive statistics of the variables used for analysis I (*N* = 667).

Name of variable	Mean	*SD*
Gender	1.56	0.496
F-economic	2.85	0.507
sm1	3.67	0.883
sm2	3.75	0.854
sm3	3.07	1.029
sm4	3.31	0.937
sm5	4.03	1.015
sm6	3.38	0.955
sm7	3.48	0.945
sm8	2.66	1.187
sm9	2.76	0.984
sm10	3.06	0.968
RA1	100.69	24.919
PTG1X	0.36	0.481

**TABLE 3:** Descriptive statistics of the variables used for analysis II (*N* = 438).

Name of variable	Mean	*SD*
Gender	1.45	0.498
F-economic	2.81	0.547
sm1	3.66	0.914
sm2	3.75	0.864
sm3	3.16	1.065
sm4	3.30	0.935
sm5	4.04	1.025
sm6	3.44	0.960
sm7	3.54	0.956
sm8	2.63	1.197
sm9	2.84	1.023
sm10	3.16	0.999
EA2	94.58	21.721
PTG1X	0.31	0.464
PTG1S	0.34	0.473

*The two samples have similar characteristics in nearly all variables. One exception was gender, meaning that we had comparable results from the two analyses.*

### Logistic Model Estimates of the Determinants of PT Participation

As showed in Table 4, for analysis I, the family economic condition could positively predict PT participation at a statistically significant level with a relatively large effect size (0.302) (Cohen, 1969), and mathematics achievement could significantly negatively predict PT participation, but with a relatively small effect size (−0.010) (Cohen, 1969).

**TABLE 4:** Results for bivariate logistic models’ PT participation for analysis I.

Independent variable	Model 1 logit coefficient	Odds ratio	Model 2 logit coefficient	Odds ratio	Model 3 logit coefficient	Odds ratio
Gender	0.169	1.184	0.177	1.194	0.169	1.184
*BA1*	−0.010***	0.990	−0.010***	0.990		
F-economic	0.302*	1.353			0.306	1.358
Sm1	−0.341***	0.711				
Sm2	0.142	1.153	0.081	1.085	0.216	1.240
Sm3	0.079	1.083	0.059	1.060	0.111	1.118
Sm4	0.013	1.013	0.011	1.011	0.033	1.034
Sm5	0.000	1.000	–0.011	0.989	0.010	1.010
Sm6	–0.136	0.873	–0.146	0.864	–0.131	0.877
Sm7	0.013	1.014	0.020	1.020	0.033	1.034
Sm8	0.177**	1.194				
Sm9	–0.027	0.973	–0.050	0.97052	0.034	1.034
Sm10	0.251**	1.285				
sm1 × F-economic			−0.081**	0.922		
sm8 × F-economic			0.071***	1.069		
sm10 × F-economic			0.101***	1.106		
Sm1 × *BA1*					−0.004***	0.996
Sm8 × *BA1*					0.002**	1.002
Sm10 × *BA1*					0.002	1.002
Constant	–1.035	0.355	–0.338	0.713	−2001***	0.135

*Significant at 10%; **significant at 5%; ***significant at 1%.

Metacognitive level could predict PT participation with different directions and a significantly large effect size. Three metacognition variables (sm1, sm8, and sm10) could predict the dependent variable with a relatively large effect size after controlling for family economic condition and mathematics achievement. Students who reported that they could clearly assess their mathematics learning and identify their class rank in mathematics learning capacity had a higher probability of not participating in PT, and those who reported that they often lack self-confidence before learning new mathematics knowledge and who reported that they often engage in self-examination regarding which parts of mathematics knowledge they had not mastered both tended to take PT across varied family economic conditions and mathematics achievement.

Three variables of metacognition: sm1, sm8, and sm10 could be moderated by the effect of family economic condition with a significant and relatively large effect. Students with a low level of ability to assess their mathematics learning and to indicate their class rank in a mathematics learning capacity would have a larger effect to select PT, when they came from rich families. In addition, when a student reported they often lacked self-confidence before learning new mathematics knowledge or often engage in self-examination to determine aspects of mathematics knowledge they have not mastered, would have a larger effect to select PT, when they came from rich families.

The moderating function of mathematics achievement was relatively small in effect size, though the effect was significant. The three active variables found covered all three sub-domains of metacognition: mathematics metacognition knowledge (sm1), mathematics metacognition experience (sm8), and mathematics metacognition monitoring (sm10).

As showed in Table 5, for analysis II, the family economic condition could positively predict participation of PT at a statistically significant level with a relatively large effect size (0.743). Students who took PT during the first year of high school were much more likely to take PT during summer vacation than students who did not take PT in the first school year, but mathematics achievement no longer has a significant effect after controlling for participation of PT in the first year of high school. Two metacognition variables (sm3 and sm8) could predict the dependent variable with a relatively large effect size.

**TABLE 5:** Results for bivariate logistic models’ PT participation for analysis II.

Independent variable	Model 1 logit coefficient	Odds ratio	Model 2 logit coefficient	Odds ratio	Model 3 logit coefficient	Odds ratio
Gender	0.275	1.317	0.276	1.318	0.275	1.316
PTG1X	2.399***	11.015	2.402***	11.040	2.425***	11.302
F-economic	0.743***	2.102			0.738***	2.091
*EA2*	0.006	1.006	0.006	1.006		
Sm1	–0.027	0.973	–0.026	0.974	–0.016	0.984
Sm2	–0.213	0.808	–0.220	0.803	–0.181	0.834
Sm3	0.381**	1.464				
Sm4	–0.238	0.788	–0.241	0.786	–0.235	0.791
Sm5	0.093	1.097	0.093	1.098	0.094	1.098
Sm6	–0.178	0.837	–0.174	0.840	–0.186	0.830
Sm7	0.100	1.105	0.108	1.114	0.116	1.123
Sm8	0.203*	1.225				
Sm9	–0.171	0.843	–0.182	0.833	–0.109	0.897
Sm10	0.125	1.134				
sm3 × F-economic			0.145***	1.156		
sm8 × F-economic			0.088**	1.092		
sm3 × *EA2*					0.002**	1.002
sm8 × *EA2*					0.002	1.002
Constant	−4.765***	0.009	−2.828***	0.059	−3.996***	0.018

*Significant at 10%; **significant at 5%; ***significant at 1%.

Students who reported the ability to set learning objectives according to different types of mathematics knowledge tended to take PT, and those who reported that they often lack self-confidence before learning new math knowledge also tended to take PT across varied family economic conditions and varied participation status of PT in the first year of high school.

It should be noted that sm8 was the common variable for different time-points in which students who reported that they often lack self-confidence before learning new mathematics knowledge would always have a high probability of taking PT.

The effect of sm3 and sm8 could be moderated by family economic condition with a significant effect size. When a student reported they often lacked self-confidence before learning new mathematics knowledge or set my learning objective accordingly, when learning different types of mathematical knowledge, they would have a larger effect to select PT, when they came from rich families.

The moderating function of mathematics achievement on the effect of metacognition was relatively small in effect size, though the effect was significant. The two active variables were found to have related sub-domains of mathematics metacognition experience (sm8) and mathematics metacognition monitoring (sm10), and the metacognition experience was a common sub-domain for both analyses.

Previous participation in PT could significantly decide future participation with an extremely large effect size (>2.000). The descriptive statistics indicated that 63.87% of students participating in PT during summer vacation all participated in PT during the two semesters of the 10th grade. These data indicated that participation in PT has a quality of inertia.

The two analyses indicated that there were differential effects of the construct on private supplementary tutoring in different time-points of high school (at the beginning of the semester and at the beginning of summer vacation).

## Discussion

The present study aimed to examine the crucial indicators of PT participation from a cluster of environmental and initial indicators. These included family economic condition and mathematics achievement (the significant indicators suggested in previous studies), and the levels of metacognition of mathematics learning, which were of immense importance in decision-making. The present study generated a specific and focused measurement for PT participation analysis, which was expected to provide more detailed information about PT participation. It identified high school students as the target population for which to provide information about students’ involvement on PT participant decision-making. In addition, the longitudinal data obtained in this study were seldom provided in previous studies. This data helped to reveal the dynamic decision-making procedure of PT participants and unearth the determinants and the related constructs of participation in PT for high school mathematics, especially the role of metacognition of mathematics learning.

The present results suggested a significant impact of family economic condition on PT participation among high school students. This is consistent with previous studies (see review in Matsuoka, 2018) and confirmed the significant prediction of environmental indicators with regard to students’ PT participation (Xue and Ding, 2009; Kim and Park, 2010). In addition, these results were expanded to the high school level. PT participation appeared to be a fundamental learning strategy for all high school students across other relevant characteristics, such as mathematics achievement, gender, and metacognitive level. Facing the pressures such as high-stake examinations. Students from rich families were more likely to participate in PT than their peers. The result was predicted in the reproduction model (Byun et al., 2018). This remarkable tendency could still be observed after controlling for previous PT participation status and mathematics achievement, which especially enrich the knowledge of the role of SES and in PT participation. These results suggested that the economic issue acts as a bottleneck in PT participation in a developing country like China, and that students tend to select PT as long as their family economic condition can support it. This result is inconsistent with previous evidence from developed countries such as Canada (Davies, 2004). In a previous study, Davies (2004) proposed that tutoring, similar to other private alternatives, would provide individualized attention as an effective competitive strategy for their children. That study suggested that students from richer families potentially tend to seek individualized attention in tutoring schools to enhance their achievement.

There were no consistent results about the impact of students’ mathematical achievement on PT participation (Wang and Guo, 2017; Byun et al., 2018; Wang et al., 2019). Zeng and Zhou (2012) suggested that the achievement-based dynamics of the decision-making procedure varied according to the areas of focus and the students’ grade level in China. Baker et al. (2001) found that in some Asian countries such as Korea and Thailand, high-achieving students were more likely than low-achieving students to use shadow education, whereas the opposite was true for many other countries. However, in this study, though students with lower mathematics achievement were significantly more likely to select mathematics PT, the effect size was relatively small. It indicated again that PT participation is a fundamental learning requirement for all high school students across other relevant characteristics, in addition to some kinds of pressure, such as examination, which was quite different from the results in other cultural setting as well as results from other school grades in Chinese setting.

For the second hypothesis, ten selected variables on the metacognition of the process of mathematics learning (covering all three sub-domains of metacognition) were included in analyses I and II. The results showed that two indicators from analysis I and three indicators from analysis II significantly impact participation with a considerable effect size as well as a different direction of effect. These results suggested an alternative domain to enrich the construct of determinants of PT and found the role of internal characteristics in the decision-making process of PT participants. Results from variable sm8, the metacognition experience variable, suggested that if the students lacked self-confidence before learning new math knowledge, they tended to choose PT for help, which indicated that the decision-making of PT participation is partly based on the analysis of students’ mathematical learning. This result could support the role of internal variables in the PT selection process. It implied that the decision-making of PT participation based on the actual need of extra tutoring of mathematics, not merely under the social pressure. And also, if the decision was proper and would benefit the students’ mathematics learning would be the focus of future studies, for example, did the students who lacked self-confidence before learning new math knowledge actually need PT and would benefit from PT.

Additional analysis found that the effect of metacognition was moderated by the family economic condition in both analyses, especially for the family economic variable. Given better family economic condition, special metacognition status (higher in some aspects and lower in others) gained a relatively larger effect size, which means that if a student got economic support, an understanding of their learning, such as “set an objective,” “often do self-examination on,” or “lack self-confidence before learning new math knowledge,” tend to make a higher probability to select PT. These results have led to further understanding about the role of metacognition in mathematics learning (Schneider and Artelt, 2010) by indicating the role of family economic condition and by indicating that students make decisions based on their own understanding of their learning.

In contrast with previous studies focusing on the parents (e.g., Davies, 2004; Liu, 2012; Liu and Bray, 2018), the present study paid more attention to the involvement of students. They suggested that, for high school students, related indicators also play important roles in the decision-making process of PT participants. The high school students tended to make PT participation decisions according to their own judgment of the learning process instead of according to their parents’ ideas.

The current longitudinal results showed that the mechanism for choosing PT tended to vary at different time-points of high school learning (beginning of high school compared to summer vacation of the first year of high school). Discussion of this learning-period-dependent variation is scarce in previous studies. Results for the middle period of high school showed that PT participation was mainly determined by PT participation in the previous school year, after controlling the related variables. It indicated that taking PT had become a chronic strategy to some extent, which indicated that, once the families decided to select PT, they tend to continue to use this strategy to help the students’ learning. It was seldom discussed in prior studies. Thus, 78.1% of students maintained their PT participation, while 68.8% of students who took PT in the first school year continued their PT participation during the following summer vacation. The results indicated that the value-added feature of PT data should be taken into consideration in the future (Sanders, 1998), and also it should be cautioned that taking PT might become the “addiction” of the students and families, which not based on students’ learning need.

In summary, the choice to participate in PT was impacted by several determinants with a moderating structure. Metacognition of mathematics learning played a role in the decision-making process across different periods of high school education, especially when the students came from wealthy families. These results indicated that PT participation was a basic learning strategy for all kinds of students, the only obstacle being a financial one. This means that more students would select PT to enhance their learning if PT was economically supported by their families.

That is, PT tended to be the only or most obvious choice for students and parents who deal with educational and social pressure. In addition, an understanding of learning, especially the metacognition experience, would enhance or reduce the motivation to participate in PT, which were moderated by family economic status.

For limitation of the current study, more control variables and the determinants variables. For example, social-psychological variables such as intrinsic motivation for mathematics, parents’ education level, educational expectation, etc. (Choi and Park, 2016), especially the internal variables of students’ mathematical learning. The quality or the characteristic of PT participation, such as the length of PT lessons, class-size, instructional content, and quality of instruction (Guill et al., 2019), should be considered. So that, future study, a more comprehensive system of variables were needed to deeply model the decision-making process, and also more qualitative studies were needed to supplement the large size quantitative analysis.

## Conclusion and Implications

The present study provided significant contribution to literature on PT participation using an integrated environment-students measure. It explored the role of metacognition and its interaction with other effective indicators, which was scarce in previous literature and constituted a structure of determinants of PT participation. The present results indicated that PT participation tended to be seen by the decision-maker to be equally important for all kinds of students, and the only barrier was a financial one. The family economic condition moderated the effect of several indices of metacognition of mathematics learning.

The analysis did not propose the best principle through which students should decide whether to participate in PT, but it illustrated a mechanism for decision-making among high school students regarding PT participation in Chinese culture. For the practical implication, educational competent department should pay more attention on the strategies which the entrepreneurs use to attract the students and their parents, and also the school teachers should actually help the students and their parents to diagnose their learning properly (help the students to enhance their ability of metacognition) and make use of PT service rationally.

Future research should focus on effectiveness of PT (Wang and Guo, 2017; Wang et al., 2019) to discuss if the families get return on their payment on the PT and what kinds of students could benefit from the PT, and help the families and students to select the PT service properly.

## Data Availability Statement

The datasets generated for this study are available on request to the corresponding author.

## Ethics Statement

Ethical review, approval were not required for the study on human participants in accordance with the local legislation and institutional requirements.

## Author Contributions

LW contributed to the conception and design of the study and organized the database. YZ and XM performed the statistical analysis. LW wrote the first draft of the manuscript and wrote sections of the manuscript. All authors contributed to the manuscript revision, read and approved the submitted version.

## Conflict of Interest

The authors declare that the research was conducted in the absence of any commercial or financial relationships that could be construed as a potential conflict of interest.

## Abbreviations

PT: private tutoring
SES: socioeconomic status
HLM: hierarchical linear modeling.
